# Cardiac Implantable Electronic Device Infections; Long-Term Outcome after Extraction and Antibiotic Treatment

**DOI:** 10.3390/idr13030059

**Published:** 2021-07-06

**Authors:** Jonas Hörnsten, Louise Axelsson, Katarina Westling

**Affiliations:** 1 Department of Cardiology, Karolinska University Hospital, 17176 Stockholm, Sweden; Jonas.Hornsten@sll.se; 2 Department of Medicine, Division of Infectious Diseases and Dermatology, Karolinska Institutet, 17177 Solna, Sweden; Axelsson.Louise@gmail.com; 3 Department of Infectious Diseases, Karolinska University Hospital, 14186 Stockholm, Sweden

**Keywords:** CIED-infections, extractions, septicemia, endocarditis, long-term survival

## Abstract

Background: The aim of the study was to examine the treatment outcome for patients with cardiovascular implantable electronic device (CIED) infections after extraction. Methods: Patients who underwent CIED extractions due to an infection at Karolinska University Hospital 2006–2015 were analyzed. Results: In total, 165 patients were reviewed, 104 (63%) with pocket infection and 61 (37%) with systemic infection. Of the patients with systemic infection, 34 and 25 patients fulfilled the criteria for definite and possible endocarditis, respectively. Complications after extraction occurred only in one patient. Reimplantation was made after a mean of 9.5 days and performed in 81% of those with pocket infection and 44.3% in systemic infection. Infection with the new device occurred in 4.6%. The mean length of hospital stay for patients with pocket infection was 5.7 days, compared to 38.6 days in systemic infection. One-year mortality was 7.7% and 22.2% in pocket infection and systemic infection, respectively. Patients with *Staphylococcus aureus* infection had a higher mortality. Conclusions: In this study, the majority of the patients had a pocket CIED infection, with a short hospital stay. Patients with a systemic infection, and *S. aureus* etiology, had a prolonged hospital stay and a higher mortality.

## 1. Introduction

In 2015, about 64,000 people were living in Sweden with cardiovascular implantable electronic devices (CIED) (The Swedish ICD and Pacemaker registry).

Infection is a major complication in CIED and the most common cause for lead extraction [1]. A study from US reported a 12% increase in CIED implantations during 2003–2006, and a 57% increase in infections [2]. The incidence of infections related to CIED has in a previous study from US been reported to be 1.9/1000 device years [3].

Risk factors for CIED infections include renal disease, diabetes mellitus, chronic obstructive pulmonary disease, corticosteroid use, heart failure, malignancy, pocket hematoma, use of anticoagulation, as well as lack of antibiotic prophylaxis before implantation [4,5]. Complications at the generator site, reimplantation, and early re-intervention may also increase the risk. 

CIED infection is presented as pocket infection or systemic infection (sepsis and endocarditis). Local infection is most common and gives such symptoms as edema, rubor and local pain [6]. *Staphylococcus* spp. have been reported as the major causative microbiological agents for CIED infections [5].

CIED infections increase morbidity and death. Cost–health economic studies have shown that CIED infections are expensive, cause prolonged hospitalization and are associated with an increased health-economic burden [7]. The use of an antibacterial envelope has recently been reported to reduce the incidence of CIED infection in a randomized multicenter study [8].

The aims of the study were to examine the differences in treatment outcome and hospitalization time for patients with CIED infection, related to type of infection, microbiological etiology and type of device. 

## 2. Materials and Methods

### 2.1. Patient Selection and Study Definitions

Karolinska University Hospital, Stockholm, is a tertiary referral hospital with 1600 beds. At the Department of Cardiology, extractions of CIED are performed. The patients are from Stockholm County as well as other counties in Sweden.

The study was retrospective and performed between 2006 and 2015, and the patients were followed-up until December 2019. Inclusion criteria were patients who underwent CIED extraction due to infection and living in Stockholm County. Exclusion criteria were patients who had the device extracted for other reasons than infection, patients from other areas than Stockholm County, and patients lacking data in their medical records.

The patients were identified from the Swedish ICD and Pacemaker Registry, and the electronic patient record, Take Care.

Information about sex, type of device, and time for extraction and reimplantation were extracted from the registry. Data from medical records were analyzed regarding device types, clinical picture, microbiological findings, length of hospital stay and antibiotic treatment. Patients were followed up for a maximum of 14 years.

The classification of CIED-infection, pocket infection (local symptoms as erythema, swelling, pain, rubor, pocket incisional drainage, and/or device exposure) or systemic infection (fever, and positive blood cultures), including infective endocarditis, was adapted from the EHRA guidelines [9] and based on data from patients’ medical records. Microbiological findings were cultures and PCR/16 S rRNA sequencing from pocket and tissue swabs, extracted leads, as well as blood cultures.

Informed consent was given by the patients to participate in the Swedish ICD and Pacemaker Registry. The study was approved by the ethical review board Karolinska Institutet, Stockholm, Sweden (2015/2289-31)

### 2.2. Extraction Procedure

Transvenous lead extractions, (TLE) were performed in our conventional pacemaker implantation laboratory by one of two experienced cardiologists trained in the procedure. Lead extractions in this study were primarily performed from the CIED pocket. The proximal end of the lead was cut, and a locking stylet was usually introduced. If simple traction was not successful passive polypropylene sheaths were then advanced coaxially over the lead body to remove the lead(s) from intravascular ingrowth of fibrotic tissue. Rarely, transfemoral vein snares were used if further difficulties were encountered or if free-floating lead ends remained. No powered sheaths (rotational sheaths or laser sheaths) were used in this study. Back-up thoracic surgeons do not attend during the lead extraction procedure but are immediately available within minutes if serious perioperative complications occur. 

### 2.3. Statistical Analysis

Summary statistics were provided as the mean. All findings were analyzed for significance and a *p*-value ≤ 0.05 was considered significant in all statistical analyses.

The collected data were analyzed in JMP version 15 and Excel. Descriptive statistics were calculated for mean age. Numerical variables were analyzed primarily with the Mann–Whitney U test, and Kruskal–Wallis H test as applicable, while categorical data in the form of the distribution of microbiological findings were analyzed with Chi-square test, and Fischer exact test when appropriate. Kaplan–Meier survival estimates were also used.

## 3. Results

Baseline data for the 165 patients who underwent transvenous lead extraction (TLE) due to an infection are described in Table 1. The mean age was 67.0 years, and 69.7% of the patients were men. Pocket infection and systemic infection occurred in 104 (63.0%) and 61 (37.0%) patients, respectively. The median follow-up was 74 months.

There were no differences between the proportions of systemic and pocket infections, in age, co-morbidity, or the proportions of women and men. 

The types of devices and previous procedures are presented in Table 2. Pacemaker extraction was the most common procedure, in women and men. Cardiac resynchronization therapy with defibrillator (CRT-D) and implantable cardioverter defibrillator (ICD) extractions were less frequent among women than men—14.7% compared to 85.3% and 11.2% compared to 88.8%, respectively (*p* = 0.002).

Complications after extraction occurred only in one patient, which was tricuspid valve damage, and surgery was performed with prosthetic replacement of the tricuspid valve.

Data for hospitalization time and length of antibiotic treatment are described in Table 3. There were no statistical differences in days of hospital stay, length of antibiotic treatment, or time until reimplantation when comparing the device types.

Echocardiography was performed in 68 patients, transesophageal echocardiography in 60 patients and transthoracic echocardiography in 8 patients. Vegetations were present in 42/68 (61.7%) of the patients examined. The locations of the vegetations were on the pacemaker electrode (n = 28), the pacemaker electrode and the tricuspid valve (n = 5), the tricuspide valve (n = 5), the aortic valve (n = 4), the mitral valve (n = 3), and others (n = 1). In total, 34 patients were classified as definite endocarditis and 25 as possible endocarditis. Patients with definite endocarditis had a longer hospital stay compared with those with possible endocarditis—43 and 31 days, respectively (*p* = 0.006)—but other factors such as length of antibiotic treatment and mortality did not differ between the two groups.

The mean time from extraction to reimplantation is described in Table 3. Reimplantation (within 3 months after removal) of the device was performed in 109/165 (66.1%) of the patients—in 82/104 (81%) and 27/61 (44.3%) for those with pocket and systemic infection, respectively. The reasons for no reimplantation were ceased indication for pacing (n = 37); 19 of these cases were patients with sick sinus syndrome transformed into chronic atrial fibrillation, severe comorbidity (n = 8), or high risk of an infection (n = 3). Other reasons were primary prophylaxis ICD with restored cardiac function. Infection in the new device during the follow-up period occurred in 5/109 patients (4.6%), which resulted in new CIED extraction in 3 of the patients.

The microbiological findings are described in Table 4. In 100% of patients with systemic infection (i.e., septicemia or endocarditis), culture and/or 16S rRNA sequencing from pockets or leads were obtained, compared to 93.3% of the patients with a local infection. In 53/165 patients (32%), 16 S rRNA sequencing was performed and yielded a positive result in 27/53 (50.9%) patients. *Staphylococcus aureus* (methicillin-resistant *S. aureus* was identified only in one patient) was the causative microorganism in 24.8% of all patients, and was identified in 44.2% of those with a systemic infection, and 13.5% of those with a local infection.

There was no significant difference in mean hospitalization time or days of antibiotic treatment if 16 S rRNA was used, compared to culture. Patients with *S. aureus* infection had a longer course of antibiotic treatment than those with an infection due to Coagulase-negative staphylococci or *Cutibacterium acnes* (*p* < 0.001).

**Figure 1:**
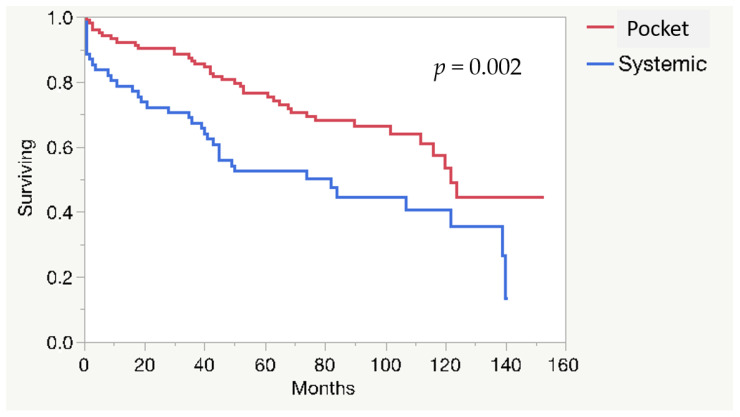
Long-term survival for patients with local and systemic CIED infection.

**Figure 2:**
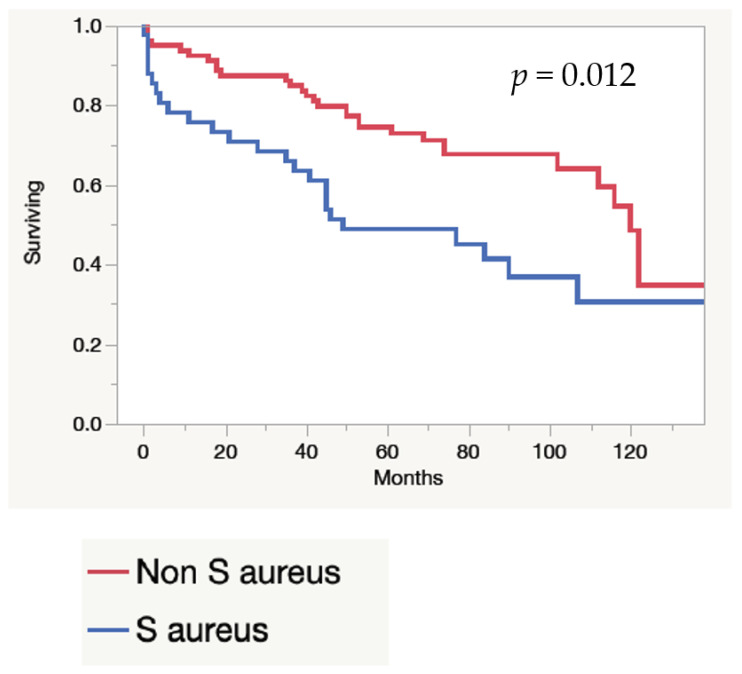
Long-term survival for CIED infections caused by *S. aureus* and non-*S aureus*.

**Figure 3:**
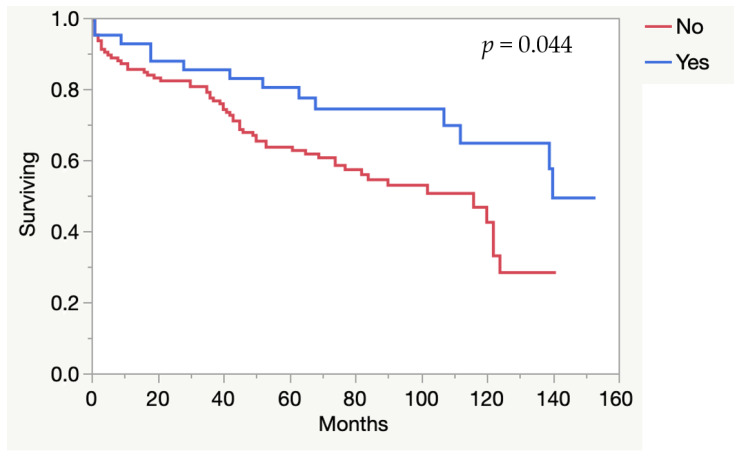
Long-term survival for patients with >2 CIED procedures performed (Yes) and ≤2 procedures (No).

The mortality rate is described in Table 5. Long-term mortality was higher among patients with systemic infection compared to pocket infection (*p* = 0.002) (Figure 1), and for *S. aureus* infection compared to non-*S. aureus* infection (*p* = 0.012) (Figure 2). In 41/165 patients (24.8%), more than two previous CIED procedures had been performed; 38% in women and 19% in men, respectively (*p* = 0.0011). Those patients had a higher long-term survival compared to those who had two or less procedures (*p* = 0.044) (Figure 3).

## 4. Discussion

In this study, 165 extractions due to CIED infection were performed, of which the majority, 69.7%, were men, something that has been presented in other studies [3,10]. There were 12,562 CIED implantations performed in Sweden in 2015, of which 35.9% were women and 64.1% men. The proportion of women (compared to men) who had a pacemaker implanted in Sweden in 2015 was 43.8%, and for ICD, CRT-P and CRT-D, the proportion of women was 19.7%, 25% and 20.8%, respectively (Swedish ICD and pacemaker registry). The results of the present study reflect these figures. In 37% of cases, the patients had a systemic infection, which is concordant with results from studies in Japan [10] and Italy [11], where the authors reported an incidence of systemic infections of 33% and 34.6%, respectively. A study from the United States [12] showed a higher incidence of systemic infections, 42%.

In our study, in patients with many previous procedures, pocket infection occurred more often, something that could be explained by repeated extractions and reimplantations, which could cause an inflammation and repeated bacterial colonization, leading to an infection. 

In the present study, positive results from microbiological cultures or PCR/16 S rRNA sequencing were found in 59.6% and 95% of patients with pocket infection and systemic infection, respectively. This is similar to the results from a study from Japan [10], in which the frequency of positive cultures derived from pocket and systemic infections was 67% and 93%, respectively.

*S. aureus* was the most common microbiological agent found in our study, dominating in patients with systemic infection. CoNS was common in patients with pocket infection. These results are consistent with other studies [3,10,13]. However, in a Danish study, CoNS was the major microbial finding both in patients with systemic infection and in those with pocket infection [14]. *C. acnes* was found in 17.3% of the patients with pocket infection in our study, and has been described as a causative agent in CIED pocket infections [15].

Patients in our study were treated for 39.7 days and 22.7 days with antibiotics for systemic infection and pocket infection, respectively. Current guidelines recommend 10–14 days of antibiotic treatment for pocket infections and 14–36 days of treatment for systemic infections after device removal [9].

In 66.1% of the patients, reimplantation of the device was performed within 3 months, which is similar to what was found in studies by Deharo and Tarakji, where 64% and 67% underwent reimplantation [16,17] during the hospitalization. Reimplantation in the present study was performed after 9.5 days for all patients, after 6.9 days for patients with pocket infection, and after 17.8 days for those with systemic infection. In the study by Deharo [16], reimplantation of the device was performed after 24 days, and most of the patients had systemic infections.

Patients with more than two CIED implants, which was more common in women than in men, had greater long-term survival. The reason for this is unclear, but could be explained by lower age, and lower degree of co-morbidity.

The guidelines recommend the removal of the CIED in patients with definite CIED infection, to prevent repeated infection [9]. Athan et al. [18] reported a one-year mortality rate in patients with CIED-endocarditis for those keeping the CIED, compared to those who underwent a removal of the CIED, of 38.1% and 19.9%, respectively.

The mean length of hospital stay was 17.9 days for all patients; 5.7 days for those with pocket infection and 38.6 days for those with systemic infection. The results are different from a recently published study from the UK, where the LOH was 8 days for patients with pocket infection and 15 days for systemic infection [7]. The patients with definite endocarditis had a longer hospital stay compared to those with possible endocarditis, maybe due to the longer wait before re-implantation of a new device. Most of the patients in our study with systemic infection received all intravenous antibiotic treatment in hospital.

One-year mortality was 13.3% for all patients, but differed between patients with a systemic infection and those with a pocket infection—22.3% and 7.7%, respectively. The result differs from those from studies in Denmark [14] and France [16], where there were no differences in long-term mortality between the groups with systemic and pocket infection; in the Danish study 14% of the patients died within 10 months [14]. In other studies published by Tarakji, 1-year mortality was 20% for all patients [12], 12% for those with pocket infection and 31% for those with a systemic infection [12], and in the study by Greenspon [1], one-year mortality was 25.3%.

Four-year mortality in the present study was 20.1% in pocket infection, and 42.6% in systemic infection. One- and four-year mortality rates were similar for patients with definite and possible endocarditis. In the French study [16], there was no difference in 5-year mortality between patients with systemic and pocket infections. In that study, there was unexpectedly no difference in long-term mortality between patients with CIED infections and those with CIED without infection, which served as a control group. Long-term survival was similar in women and men in our study, which differs from the result of a study from the Unites States [19], wherein women had better long-term mortality rates than men.

## 5. Conclusions

In this study most of the patients had a pocket CIED infection. The complication rate after extraction was low. Those with a systemic infection, mainly caused by *S. aureus*, had a prolonged hospital stay, and higher mortality. Patients with definite and possible endocarditis had similar long-term mortality. 

## Figures and Tables

**Table 1:** Baseline data for 165 patients who underwent removal for CIED infection in 2006–2015 at Karolinska University Hospital.

	Pocket Infection	Systemic Infection	All Infections	*p*-Value
**Number of patients**	104	61	165	
Mean age (y) ± SD	66.9 ± 15.0	69.8 (±12.4)	67.0 ± 14.1	
Sex female n (%)	33 (31.7%)	17 (27.9%)	50 (30.3%)	0.601
Heart failure n (%)	36 (34.6%)	27 (44.3%)	63 (38.1%)	0.219
Coronary artery disease n (%)	26 (25.0%)	19 (31.1%)	45 (27.2%)	0.394
Valvular heart disease n (%)	21 (20.2%)	13 (21.3%)	34 (20.6%)	0.864
Hypertension n (%)	20 (19.2%)	8 (13.1%)	28 (17.0%)	0.310
CABG n (%)	16 (15.4%)	12 (19.7%)	28 (16.9%)	0.47
Diabetes mellitus n (%)	11 (10.6%)	12 (19.6%)	23 (13.9%)	0.11

Abbreviations: CIED = cardiovascular implantable electronic devices, CABG = Coronary artery bypass graft surgery

**Table 2:** Type of device and previous procedures in 165 patients who performed extraction due to CIED infection.

	Pocket Infection	Systemic Infection	All Infections	*p*-Value
**Number of patients**	104	61	165	
Type of device				
PM	53 (50.9%)	36 (59.1%)	89 (53.9%)	0.615
CRT-D	21 (20.2%)	13 (21.3%)	34 (20.6%)	0.86
ICD	19 (18.3%)	8 (13.1%)	27 (16.4%)	0.39
CRT-P	11 (10.6%)	4 (6.6%)	15 (9.1%)	0.38
De novo implantation	20 (19.2%)	30 (49.2%)	40 (30.3%)	0.004
Reimplantation	84 (80.8%)	31 (50.8%)	115 (69.7%)	0.004
More than 2 CIED procedures	29 (27.8%)	12 (19.7%)	41 (24.8%)	0.23

Abbreviations: CIED = cardiovascular implantable electronic devices, PM = pacemaker, ICD = implantable cardioverter defibrillator, CRT-D = cardiac resynchronization therapy with defibrillator, CRT-P = cardiac resynchronization therapy with pacemaker.

**Table 3:** Length of hospitalization, days of antibiotics treatment, and days until re-implantation in 165 patients with CIED infection.

	Pocket Infection (n = 104)	Systemic Infection (n = 61)	All Infections (n = 165)	*p*-Value
Length of hospitalization	5.7 (±5.9)	38.6 (±15.7)	17.9 (±19)	<0.001
Days to reimplantation	6.9 (±14.6)	17.8 (±17.9)	9.5 (±16)	0.006
Days on antibiotic treatment	22.7 (±22.6)	39.7(±16.02)	29.9 (±21.9)	<0.001

Days in mean ± SD. Abbreviations: CIED = cardiovascular implantable electronic devices.

**Table 4:** Microbiological findings in 165 patients with CIED removal due to infection.

	Pocket Infection (n = 104)	Systemic Infection (n = 61)	All Infections (n = 165)	*p*-Value
*Staphylococcus aureus*	14 (13.5%)	27 (44.2%)	41 (24.8%)	<0.001
Coagulase-negative staphylococci	23 (22.1%)	15 (24.5%)	38 (23.0%)	0.7
*Cutibacterium acnes*	18 (17.3%)	4 (6.5%)	22 (13.3%)	0.028
*Streptococcus* spp.	0	6 (9.8%)	6 (3.6%)	-
Other	7 (6.7%)	6 (9.8%)	13 (7.9%)	0.47
Negative culture or PCR	35 (33.5%)	3 (4.9%)	38 (23.0%)	0.0023
Not performed	7 (6.7%)	0	7 (4.2%)	-

Data expressed in number (%). Abbreviations: CIED = cardiovascular implantable electronic devices, *S. aureus* = *Staphylococcus aureus*, CoNS = coagulase-negative staphylococci, *C. acnes* (*Cutibacterium acnes*), Other = identified microbe other than above mentioned, Negative = no findings in cultures or 16 S rRNA/PCR, None = no bacterial cultures or PCR testing performed.

**Table 5:** Long-term mortality for patients who underwent an extraction due to CIED infection.

	Pocket Infection	Systemic Infection	All Infections	*p*-Value
Number of patients	104	61	165	
30—day mortality	0	3 (17%)	3 (1.8%)	-
1—year mortality	8 (7.7%)	14 (22.3%)	22 (13.3%)	0.006
4—year mortality	21 (20.1%)	26 (42.6%)	47 (28.4%)	0.002

Data expressed in number (%). Abbreviations: CIED = cardiovascular implantable electronic devices.

## Data Availability

The data supporting the findings in the study will be available on request from the corresponding author.

## References

[1] Greenspon A.J., Patel J.D., Lau E., Ochoa J.A., Frisch D.R., Ho R.T., Pavri B.B., Kurtz S.M. (2011). 16-year trends in the infection burden for pacemakers and implantable cardioverter-defibrillators in the United States 1993 to 2008. J. Am. Coll. Cardiol..

[2] Voigt A., Shalaby A., Saba S. (2010). Pacing Continued rise in rates of cardiovascular implantable electronic device infections in the United States: Temporal trends and causative insights. Pacing Clin. Electrophysiol..

[3] Uslan D.Z., Sohail M.R., St Sauver J.L., Friedman P.A., Hayes D.L., Stoner S.M., Wilson W.R., Steckelberg J.M., Baddour L.M. (2007). Permanent pacemaker and implantable cardioverter defibrillator infection: A population-based study. Arch. Intern. Med..

[4] Polyzos K.A., Konstantelias A.A., Falagas M.E. (2015). Risk factors for cardiac implantable electronic device infection: A systematic review and meta-analysis. Europace.

[5] Arnold C.J., Chu V.H. (2018). Cardiovascular Implantable Electronic Device Infections. Infect. Dis. Clin. N. Am..

[6] Sohail M.R., Uslan D.Z., Khan A.H., Friedman P.A., Hayes D.L., Wilson W.R., Steckelberg J.M., Stoner S., Baddour L.M. (2007). Management and outcome of permanent pacemaker and implantable cardioverter-defibrillator infections. J. Am. Coll. Cardiol..

[7] Ahmed F.Z., Fullwood C., Zaman M., Qamruddin A., Cunnington C., Mamas M.A., Sandoe J., Motwani M., Zaidi A. (2019). Cardiac implantable electronic device (CIED) infections are expensive and associated with prolonged hospitalisation: UK Retrospective Observational Study. PLoS ONE.

[8] Tarakji K.G., Mittal S., Kennergren C., Corey R., Poole J.E., Schloss E., Gallastegui J., Pickett R.A., Evonich R., Philippon F. (2019). Antibacterial envelope to prevent cardiac implantable device. N. Engl. J. Med..

[9] Blomström-Lundqvist C., Traykov V., Erba P.A., Burri H., Nielsen J.C., Bongiorni M.G., Poole J., Boriani G., Costa R., Deharo J.C. (2020). European Heart Rhytm Association (EHRA) International Consensus Document on how to prevent, diagnose, and treat cardiac implantable electronic device infections. Europace.

[10] Fukunaga M., Goya M., Nagashima M., Hiroshima K., Yamada T., An Y., Hayashi K., Makihara Y., Ohe M., Ichihashi K. (2017). Identification of causative organism in cardiac implantable electronic device infections. J. Cardiol..

[11] Bongiorni M.G., Tascini C., Tagliaferri E., Di Cori A., Soldati E., Leonildi A., Zucchelli G., Ciullo I., Menichetti F. (2012). Microbiology of cardiac implantable electronic device infections. Europace.

[12] Tarakji K.G., Wazni O.M., Harb S., Hsu A., Saliba W., Wilkoff B.L. (2014). Risk factors for 1-year mortality among patients with cardiac implantable electronic device infection undergoing transvenous lead extraction: The impact of the infection type and the presence of vegetation on survival. Europace.

[13] Carrasco F., Anguita M., Ruiz M., Castillo J.C., Delgado M., Mesa D., Romo E., Pan M., Suárez de Lezo J. (2016). Clinical features and changes in epidemiology of infective endocarditis on pacemaker devices over a 27-year period (1987–2013). Europace.

[14] Ihlemann N., Møller-Hansen M., Salado-Rasmussen K., Videbaek R., Moser C., Iversen K., Bundgaard H. (2016). CIED infection with either pocket or systemic infection presentation—Complete device removal and long-term antibiotic treatment; long-term outcome. Scand. Cardiovasc. J..

[15] El Rafei A., Desimone D.C., Sohail M.R., Desimone C.V., Steckelberg J.M., Wilson W.R., Baddour L. (2016). Cardiovascular Implantable Electronic Device Infections due to Propionibacterium Species. Pacing Clin. Electrophysiol..

[16] Deharo J.C., Quatre A., Mancini J., Khairy P., Le Dolley Y., Casalta J.P., Peyrose E., Prévôt S., Thuny F., Collart F. (2012). Long-term outcomes following infection of cardiac implantable electronic devices: A prospective matched cohort study. Heart.

[17] Tarakji K.G., Chan E.J., Cantillon D.J., Doonan A.L., Hu T., Schmitt S., Fraser T.G., Kim A., Gordon S.M., Wilkoff B. (2010). Cardiac implantable electronic device infections: Presentation, management, and patient outcomes. Heart Rhythm..

[18] Athan E., Chu V.H., Tattevin P., Selton-Suty C., Jones P., Naber C., Miró J.M., Ninot S., Fernández-Hidalgo N., Durante-Mangoni E. (2012). Clinical characteristics and outcome of infective endocarditis involving implantable cardiac devices. JAMA.

[19] Sohail M.R., Henrikson C.A., Braid-Forbes M.J., Forbes K.F., Lerner D.J. (2013). Comparison of mortality in women versus men with infections involving cardiovascular implantable electronic device. Am. J. Cardiol..

